# Lentivirus‐mediated shRNA interference of ghrelin receptor blocks proliferation in the colorectal cancer cells

**DOI:** 10.1002/cam4.723

**Published:** 2016-07-27

**Authors:** An Liu, Chenggang Huang, Jia Xu, Xuehong Cai

**Affiliations:** ^1^ Department of General SurgeryThe First People Hospital of Yueyang39 Dong mao ling RoadYueyangHunan325000China

**Keywords:** Colorectal cancer, ghrelin, growth hormone secretagogue receptor, proliferation, PTEN

## Abstract

Ghrelin, an orexigenic peptide, acts via the growth hormone secretagogue receptor (GHSR) to stimulate the release of growth hormone. Moreover, it has a range of biological actions, including the stimulation of food intake, modulation of insulin signaling and cardiovascular effects. Recently, it has been demonstrated that ghrelin has a proliferative and antiapoptotic effects in cancers, suggesting a potential role in promoting tumor growth. However, it remains unknown whether GHSR contributes to colorectal cancer proliferation. In this study, the therapeutic effect of lentivirus‐mediated short hairpin RNA (shRNA) targeting ghrelin receptor 1a (GHSR1a) was analyzed in colorectal cancer cell line SW480 both in vitro and in vivo. Our study demonstrated that ghrelin and GHSR1a are significantly upregulated in cancerous colorectal tissue samples and cell lines. In vitro, human colorectal cancer cell line SW480 with downregulation of GHSR1a by shRNA showed significant inhibition of cell viability compared with blank control (BC) or scrambled control (SC) regardless of the application of exogenous ghrelin. Furthermore, GHSR1a silencing by target specific shRNA was shown capable of increasing PTEN, inhibiting AKT phosphorylation and promoting the release of p53 in SW480 cells. In addition, the effects of GHSR1a knockdown were further explored in vivo using colorectal tumor xenograft mouse model. The tumor weights were decreased markedly in GHSR1*α* knockdown SW480 mouse xenograft tumors compared with blank control or negative control tumors. Our results suggested that the expression of GHSR1a is significantly correlated with the growth of colorectal cancer cells, and the GHSR1a knockdown approach may be a potential therapy for the treatment of colorectal cancer.

## Introduction

Colorectal cancer is a common visceral tumor that has been treated primarily merely by surgery for decades 1, 2. Currently, using preoperative radiotherapy alone or in combination with chemotherapy is widely accepted as a means to improve disease‐free survival (DFS) and overall survival (OS) 3, 4. It remains a priority to identify potential biomarkers that could predict recurrence, DFS and OS. Moreover, no insight into the molecular events responsible for tumor proliferation, invasion and other malignant biological properties was given by the majority of prognostic factors 5, 6.

Ghrelin, an orexigenic peptide, is primarily produced and secreted by the gastrointestinal tract. It is implicated in a wide range of physiological as well as pathophysiological activities including the control of food intake and metabolism 7, 8. Recent studies have reported that ghrelin can modulate cell physiology and pathophysiology based on an autocrine mechanism through widely expressed unique class of receptors, which are known as Growth Hormone Secretagogue Receptors (GHSRs). These receptors have two known subtypes, GHSR1a and GHSR1b 9, 10, 11. The GHSR‐1a has been found distributed in vascular endothelium, myocardium, and monocytes 12. Cellular signals carried by ghrelin are transduced by GHSR‐1a 13. In somatotroph cells, the activation of GHSR‐1a by ghrelin induces ghrelin release through enhanced phospholipase C activity, protein kinase C, and intracellular calcium mobilization 14. In addition to regulating normal growth and differentiation, one relatively newer but exciting dimension is the role of ghrelin in malignant cell proliferation as well as invasion. Ghrelin and/or GHSR has been implicated in the maintenance as well as progression of several human malignant tumors, including esophageal 15, gastric 16, lung 17, and endometrial cancer 18. Existing studies of the mechanistic machinery revealed that both exogenous and endogenously produced ghrelin could account for the increased proliferation in several malignant cancer cells in vitro 15, 16, 17, 18. Although few authors have arguably showed that ghrelin axis exert antiproliferative role in malignant neoplasms 19, but the weight of evidence favors the proliferative role of ghrelin and/or GHSR 15, 16, 17, 18. However, the relationship between ghrelin axis and colorectal cancer still remains unclear. In this study, we explored the role of ghrelin and GHSR1a in the progression of colorectal malignancy using short‐hairpin (sh) RNA‐mediated GHSR1a gene silencing in cell culture as well as in vivo level.

## Materials and Methods

### Patients and tissue specimens

The study protocol was approved by the ethics committee of the First People Hospital of Yueyang. One hundred and fifty patients (87 men and 63 women) with a histologically proven localized adenocarcinoma of the colorectum, treated by curative resection at the department of general surgery of the First People Hospital of Yueyang in China between 2013 and 2015 were included in this study. The age of the patients ranged from 39 to 74 with a mean of 56 ± 6 years. All the 150 colorectal cancer patients gave written consent to participate in the study. No patients had received chemotherapy or radiotherapy prior to surgery. Other exclusion criteria included acute or chronic infection, dysphagia, congestive heart failure, chronic obstructive pulmonary disease (COPD), eating disorders, gastrectomy and cirrhosis.

### Cell culture

Human well‐differentiated colon cancer cell line Caco‐2 and poorly differentiated colon cancer cell line SW480 were purchased from American Type Culture Collection (ATCC, Rockville, MD). Nonmalignant colon epithelial cell line NCM460 was preserved in our laboratory. The cells were cultured in Dulbecco's Modified Eagle Medium (DMEM) containing 10% fetal bovine serum (FBS), 0.5% penicillin‐streptomycin and 1% glutamine at 37°C with 5% CO_2_. Cells in the logarithmic growth phase (~90% confluence) were selected for the experiments.

### Reverse transcription‐polymerase chain reaction analysis

Total RNAs extraction was performed using Trizol Reagent (Life Technologies, Carlsbad, CA) according to the manufacturer's instructions. RNA concentrations were determined by spectrophotometry. cDNA was produced according to the protocol for PrimeScript^™^ RT Reagent (TaKaRa, Shiga, Japan). Each reverse transcript was amplified with GAPDH as an internal control. For GAPDH amplification, there were a total of 20 PCR cycles. The PCR products were electrophoresed in a 2% agarose gel and visualized by staining with ethidium bromide. The primer sequences for human ghrelin were as follows: forward 5′‐TGAGCCCTGAACACCAGAGAG‐3′ and reverse 5′‐AAAGCCAGATGAGCGCTTCTA‐3′. The same forward primer was used for human GHS‐R1a: 5′‐TCGTG GGTGCCTCGCT‐3′.Reverse primers for human GHS‐R1a was 5′‐CACCACTACAGCCAGCATTTTC‐3′. GAPDH primers were forward 5′‐AACGGATTTGGTCGTATTGGG‐3′; antisense, 5′‐TCGCTCCTGGAAGATGGTGAT‐3′. The gene expression levels obtained were normalized by mRNA expression of GAPDH. The relative mRNA expression was then presented in relation to the control.

### Western blot analysis

Cells were washed with ice‐cold PBS and lysed in NP 40 lysis buffer for three times (20 mmol L^−1^ Tris‐HCl (pH 7.4), 100 mmol L^−1^ NaCl, 1% NP40, 0.5% sodium deoxycholate, 5 mmol L^−1^ MgCl_2_, 0.1 mmol L^−1^ phenylmethylsulfonyl fluoride, and 10 mg mL^−1^ of protease inhibitor mixture). Protein was extracted using Mammalian Protein Extraction Reagent (Pierce Inc., Rockford, IL), whereas its concentration was determined by BCA (Pierce) assay. Total proteins (20–40 *μ*g) extracted from each sample were electrophoresized in 8 % SDS‐PAGE gel, and transferred to a nitrocellulose membrane. The membranes were blocked in 5% nonfat milk as well as probed with the primary antibodies as indicated overnight at 4°C. Afterward, they were probed again with respective secondary antibodies. Band signals were visualized using an enhanced chemiluminescence kit (Pierce, Minneapolis, MN). The same membrane was reprobed with the anti‐*β*‐actin antibody, which was used as the internal control.

The polyclonal goat anti‐human GHSR1a primary antibody and polyclonal rabbit anti‐human ghrelin primary antibody were obtained from Santa Cruz. Anti‐*β*‐actin and horseradish peroxidase secondary antibody were obtained from Abcam.

### Cell viability

SW480 cells were seeded in each well of the 96‐well culture plates (1000 cells per well). After overnight incubation, medium was removed and replaced with fresh culture medium plus equal amounts of different conditioned medium. After 48 h of incubation, the supernatant was discarded and 10 *μ*L CCK‐8 (cell counting kit‐8); solution (Dojin Laboratory, Kumamoto, Japan) was added. Subsequently, the cells were incubated at 37°C for 60 min, whereas the absorbance was measured at 490 nm using a microplate spectrophotometer (Bio‐Tek, Winooski, VT, USA).

### Lentiviral silencing of GHSR1a in colorectal cancer cell line shRNA design and vector preparation

In accordance with a published sequence of human GHSR1a (NM 198407), certain shRNAs specifically targeting human GHSR1a were designed to knockdown its expression. In parallel, a negative control was projected so that any gene profile changes could be discounted through lentiviral delivery approaches. There were 19 nucleotides (nt) of the target sequence in the short hairpin RNA (shRNA) expression cassette before the loop sequence (TTC AAGAGA). The shRNA sequences are GHSR1a (sense: 5′‐TGAGAAAGCTCTCCACTCTGTTCAAGAGACAGAGTGGAGAGCTTTCTCTTTTTTC‐3′; antisense: 5′‐ACTCTTTCGAGAGGTGAGACAAGTTCTCTGTCT CACCTCTCGAAAGAGAAAAAAGAGCT‐3′ and scrambled (sense: 5′‐TAACTAGTAACGGCTGCTCCTTCAAGAGAGGAGCAGCCGTTACTAGTTTTTTTTC‐3′; antisense: 5′‐ATTGATCATTGCCGACGAGGAAGTTCTCTCCTCGTCGGCAATGATCAAAAAAAAGAGCT‐3′. After the commercial synthesis (Sigma‐Aldrich, St. Louis, MO, USA) of the shRNA cassettes as well as their supplementary sequences, they were then annealed for 5 min in a buffer that was heated to 95°C and then cooled down to room temperature. At the Xhol and Hapl sites, the generated double‐stranded oligo DNA was cloned into plasmid. In addition, enhanced green fluorescent protein was expressed by the pLL3.7 plasmid. The Version 3.1 protocol of ABI PRISM BigDye Terminator Cycle Sequencing Kit (Life Technologies) was used by the Australian Genome Research Facility (Brisbane, Australia) to confirm the insert via both DNA sequencing as well as restricted enzyme digestion.

### Lentiviral packaging and infection of target cell line

Following the user manual, Escherichia coli was used to amplify the packaging plasmid and the pLL3.7, while they were also purified by W/Endo‐free Invitrogen Maxi‐Prep Kits (Life Technologies). The HEK‐293T packaging cell line was transfected with pLL3.7 and the lentiviral packaging plasmids, including pRSVRev, pMD.G (contains VSV.G gene), as well as pMDLgpRRE. On the following day, the medium was replaced with 10 mL of fresh DMEM. After another 48 h, supernatant was collected and filtered. Also, the vivaspin 22‐mL concentrators were used to concentrate the viral supernatant into 40–50 times by ultracentrifuging at the speed of 22,000 *g* for a duration of 90 min at 4°C. The lentiviral stocks were stored in small aliquots at −80°C for titration and cell infection. After being seeded into six‐well plates (100, 000 cells per well), the SW480 cells were then cultured overnight. Lentiviral stocks were diluted with DMEM‐F‐12 medium (Life Technologies) that contained hexadimethrine bromide (final concentration 8 g/mL). Subsequently, the mixture was added to the cells to incubate for 24 h at 37°C. After a 24‐h infection, the medium was replaced with 10 mL of fresh DMEM‐F‐12 for another 48 h. Finally, the cells were harvested for the cell line selection of stable transfection, whereas a fluorescence microscope was in use and detected cells that express green fluorescence.

### In vivo tumorigenesis

In vivo experiments were conducted as described previously 18. Briefly, we used male athymic BALB/c nu/nu mice (4–6 weeks old) that had been maintained in the standard mouse plexiglass cages in a room maintained at constant temperature as well as humidity under 12 h light and darkness cycles. The 1 × 10^7^ logarithmically growing cells, which were divided into three groups, involving (blank control, BC) SW480 cells transfected with culture media; (SC) SW480 cells transfected with scrambled shRNA; (knockdown, KD) SW480 cells transfected with GHSR1a shRNA, were injected subcutaneously into the midabdominal, respectively. After 40 days of observation, the mice were killed while the tumors were surgically excised neatly and weighed. One part of the tissue was fixed in formalin and another part was frozen in liquid nitrogen.

### Immunolocalization of PTEN and Ki‐67 in tumor samples

Xenograft tumor samples were embedded in paraffin and fixed with paraformaldehyde. After being washed in PBS, the slides were blocked with protein block solution (DakoCytomation) to obstruct endogenous peroxidase activity. Samples were incubated with the following primary antibodies: rabbit polyclonal anti‐PTEN (Sigma‐Aldrich) or rabbit polyclonal anti‐Ki67 (Abcam, Cambridge, England). The dilutions of antibodies that were used for immunohistochemical localization of ghrelin, GHSR1a, PTEN, and Ki‐67 are 1:100, 1:200, 1:100, and 1:500, respectively. Normal host serum was used for negative controls, followed by staining with appropriate HRP‐conjugated secondary antibodies. The peroxidase was visualized with 3‐3'‐diaminobenzidinetetrahydrochloride solution and then counterstained with a weak hematoxylin solution stain. The stained slides were dehydrated and visualized on an Olympus microscope (Olympus, Shinjuku, Japan) by an investigator that was blinded to the xenograft tumor groups. The stained sections were counted in 10 random views at 200 × magnification. In each view, the grayscale intensity was measured at 20 randomly selected points and averaged. The value of integrated option density (IOD) was proportional to the PTEN protein level, and the proliferation index was expressed as Ki‐67‐positive cells/total cells in the view ×100%.

### Statistical analysis

Data were represented as mean ± S.D for the absolute values or percentages of controls. Statistical differences were evaluated by one‐way analysis of variance (ANOVA) followed by LSD multiple comparisons tests through using SPSS software (version 17.0, SPSS, Inc., Chicago, IL, USA). A value of *P *<* *0.05 was considered statistically significant.

## Results

### Analysis of ghrelin and GHSR1a in colorectal cancer cell lines and clinical specimens

**Figure 1:**
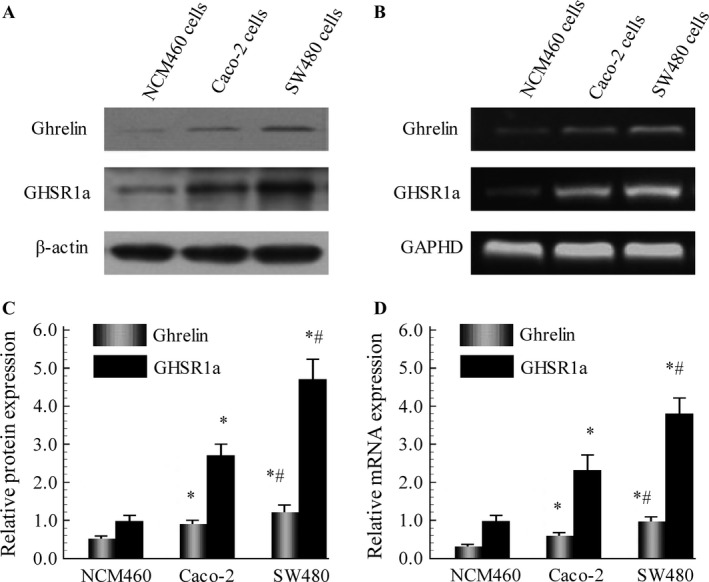
Levels of ghrelin and GHSR1a were significantly higher in malignant Caco‐2 and SW480 cells than in normal human colonocytes using Western blot (A) as well as RT‐PCR (B). C and D: Semiquantitative analysis was performed on ghrelin and GHSR1a levels. Western immunoblotting for *β*‐actin protein was performed as loading control representative. RT‐PCR for GAPDH was used as a control for amplification. Results are reported as the mean ± SD of four independent experiments. *P <* *0.05 versus NCM460 group, ^#^ *P *<* *0.05 versus. Caco‐2 group.

**Figure 2:**
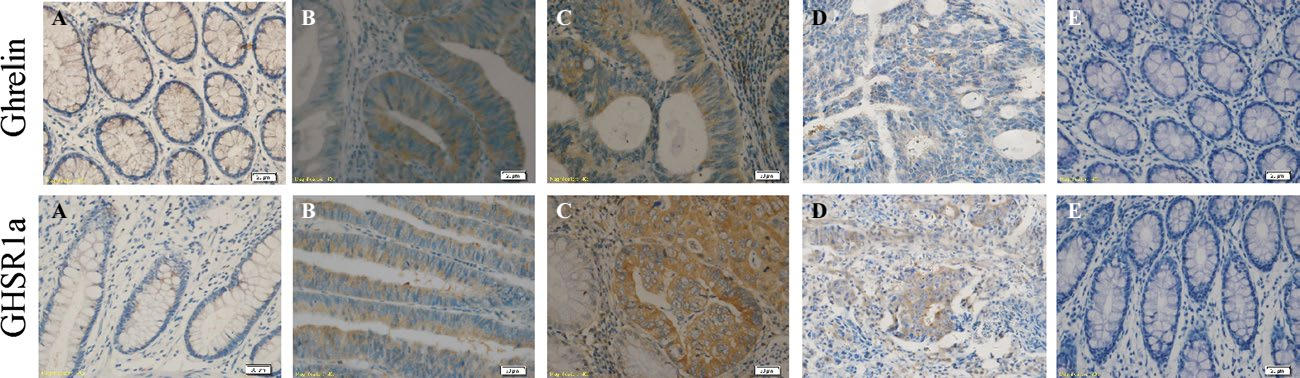
Representative immunohistochemical localization of ghrelin (upper row) and GHSR1a (lower row) in normal colorectal tissues (A), well (B), moderately (C), and poorly differentiated colorectal cancer tissues (D). E: Human normal colorectal tissue sections incubated without primary antibody were used as negative control. Scale bars, 20 *μ*m.

Initial experiments focused on the characterization of ghrelin and GHSR1a in colorectal cancer cells. We examined the relative expression levels of ghrelin‐GHSR system in colorectal cancer cell lines Caco‐2 and SW480, along with NCM460, a non‐malignant colon epithelial cell line. Western blot analysis showed that ghrelin and GHSR1a protein levels were barely detectable in NCM460 cells. It also demonstrated that such levels in Caco‐2 and SW480 cells were significantly higher than in NCM460 cells (Fig. 1A). Furthermore, the changes in ghrelin and GHSR1a mRNA levels revealed by Reverse transcription‐polymerase chain reaction (RT‐PCR) analysis in normal and malignant colorectal cells were similar to the results of Western blot (Fig. 1B). As the expression of GHSR1a in SW480 cells was markedly over‐expressed in contrast to Caco‐2 cells, SW480 cells was chosen in the subsequent experiments. For the immunohistochemical detection of ghrelin‐GHSR system in colorectal malignant cell behavior at in vivo level, we analyzed the expression of ghrelin and GHSR1a in normal as well as malignant colorectal tissue samples. Positive immunohistochemical staining for ghrelin and GHSR1a were present in cytoplasmic compartment in all of the tissue samples. Ghrelin and GHSR1a expression were significantly enhanced in malignant tissue samples in comparison with normal colorectal tissues. However, there was no correlation between the intensity of ghrelin or GHSR1a immunostaining and tumor grades (Fig. 2).

### GHSR1a‐shRNA significantly inhibited GHSR1a mRNA and protein expression in SW480 cells

**Figure 3:**
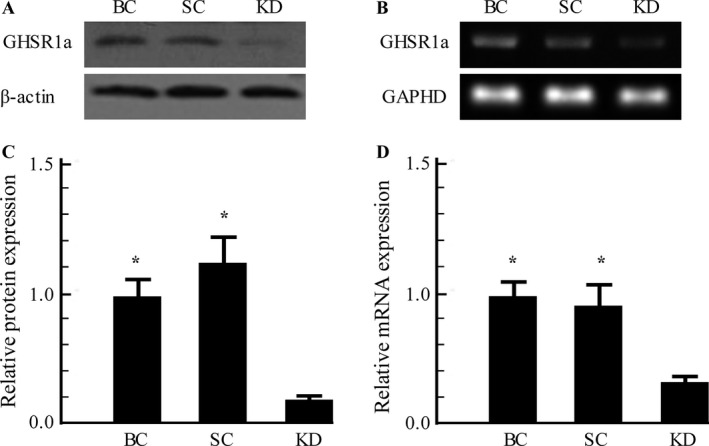
The production of stable GHSR1a knockdown cells. (A) Knockdown of GHSR1a protein was assayed by western blot analysis. (B) Decrease in GHSR1a mRNA Level in SW480 cells was detected by RT‐PCR. Similar results were obtained in three independent experiments. *P <* *0.05 versus KD group. BC, blank control cells; SC, scrambled control cells; KD, GHSR1a Knockdown cells.

To determine the effect of GHSR1a‐knockdown (KD) on the expression of GHSR1a in SW480 cells, mRNA and protein levels were analyzed by RT‐PCR along with western blot analysis. As shown in Figure 3, the transduction of GHSR1a‐shRNA resulted in a considerable decrease in the levels of GHSR1a protein (Fig. 3A) and mRNA (Fig. 3B) compared with that of SC group cells or BC group cells (*P *<* *0.05), whereas the expression of GHSR1a was similar between SC group cells and BC group cells at the transcriptional as well as translational levels.

### GHSR1a knockdown inhibits the growth of SW480 cells

**Figure 4:**
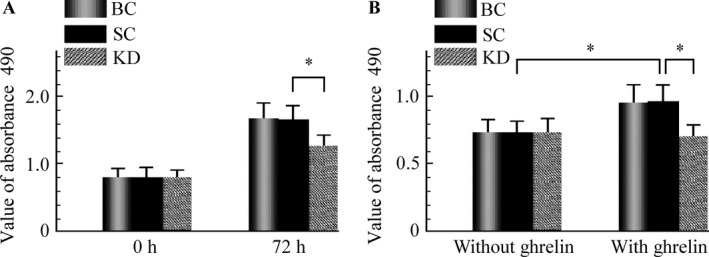
Effect of GHSR1a short hairpin RNA (shRNA) on the growth of SW480 cells. (A) SW480 cells were transduced with culture medium (BC), SC, and GHSR1a shRNA (KD) were monitored for 72 h, and cell viability was measured by (CCK‐8 assay). (B) Effect of exogenous ghrelin on the growth of SW480 cells. Each experiment was repeated triplicate. *P <* *0.05. BC, blank control; CCK‐8 assay cell counting kit‐8; SC, scrambled control, KD, knockdown.

Secondly, the effects of GHSR1a shRNA on the growth of SW480 cells were evaluated. As shown in Figure 4A, in the absence of exogenous ghrelin treatment, the viability of cells was significantly inhibited by (32.24 ± 3.67) % after 72 h of transfection with GHSR1a shRNA in comparison with the blank or scrambled controls (*P *<* *0.05). Furthermore, to explore the importance of GHSR1a in the cell growth of colorectal cancer cells stimulated with ghrelin, SW480 cells were exposed to exogenous acylated ghrelin (1000 nmol) for 72 h and cell viability was examined by CCK‐8 assay. The CCK‐8 assay indicated that after being cultured with ghrelin, the cell viability was significantly increased by (31.57 ± 3.61) % in SW480 cells. However, there was no increase in viability in GHSR1a shRNA transduced cells with ghrelin stimulation (Fig. 4B).

### GHSR1a shRNA upregulated PTEN and inhibited the PI3K/AKT pathway

**Figure 5:**
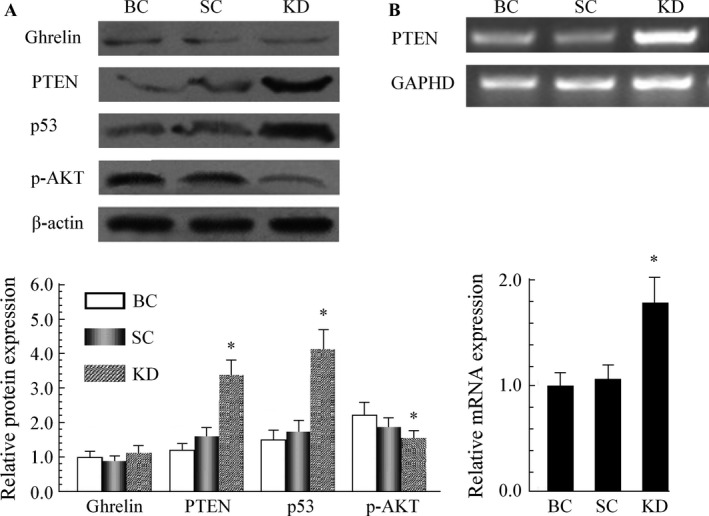
GHSR1a shRNA upregulated PTEN and inhibited the PI3K/AKT pathway in SW480 cells. (A) Western blot analysis of PTEN, p‐AKT, and p53 after transducted with culture medium (BC), SC, and GHSR1a shRNA (KD). (B) The PTEN mRNA was detected by RT‐PCR assay. Data were derived from three experiments with six replicates. *P <* *0.05 versus SC group. shRNA, short hairpin RNA; BC, blank control; SC, scrambled control; KD, knockdown.

As shown in Figure 5A, the transduction of GHSR1a shRNA had no impact on the expression of ghrelin in SW480 cells. To investigate whether the regulation of PI3K/AKT survival pathway is involved in the GHSR1a shRNA‐induced antiproliferation activity on SW480 cells, the expression of PTEN and its regulatory proteins in SW480 cells were measured by Western blot. The result indicated that the downregulation of GHSR1a increased the protein level of PTEN, whereas the p‐AKT and p53 levels were decreased and increased, respectively, owing to the effects of GHSR1a shRNA transduction. Next, we further investigated whether GHSR1a shRNA increases PTEN at transcription level in SW480 cells. The expression of PTEN mRNA was increased by 1.8 fold after being transfected with GHSR1a shRNA (Fig. 5B). Taken together, such data suggested that the regulation of PTEN/PI3K/AKT pathway is associated with GHSR1a‐induced proliferation in SW480 cells.

### GHSR1a shRNA inhibits tumor growth in nude mice

**Figure 6:**
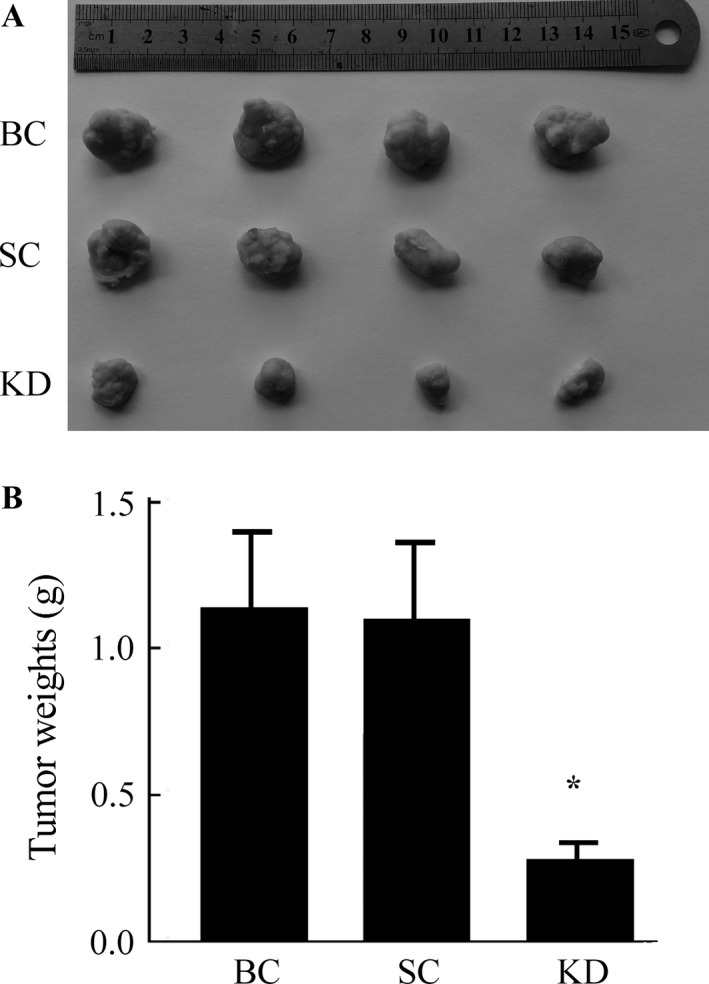
The effect of GHSR1a knockdown on in vivo growth of tumor xenograft. (A) Necropsy photographs of mice bearing colorectal tumors in mice showing therapeutic benefit of GHSR1a knockdown. (B) Showed the mean weight of each group. Similar results were obtained in three independent experiments. *P <* *0.05 versus blank control,(BC) or scrambled control (SC) groups.

To confirm the tumorigenic effect of GHSR1a in vivo, xenograft model was performed to compare the tumorigenesis of SW480 cells with or without GHSR1a shRNA transfection. Subcutaneous tumor nodes of different groups became palpable after 15 days of transplantation. The antitumor efficacy of GHSR1a KD was determined by considering mean tumor weight immediately following euthanization. As shown in Figure 6A, The final tumor weights showed significant decrease in the KD group compared with BC or SC groups (*P *<* *0.05). In addition, the tumor weight in BC group was not as statistically significant as the SC group (Fig. 6B).

### Tumor immunohistochemistry in vivo

**Figure 7:**
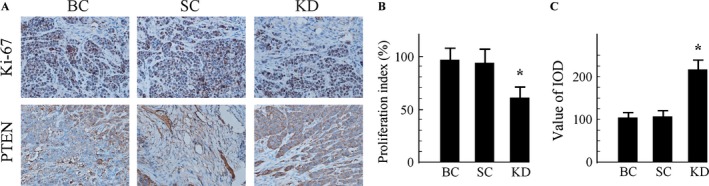
Immunohistochemistry for Ki‐67 and PTEN in tumor tissue sections (Original magnification ×200). (A) Representative images of Ki67 (upper) and PTEN (lower) staining of xenograft tumors. (B) Ki‐67‐positive nuclei were counted, and data were represented as means ± SD. (C) The relative expression of PTEN protein was significantly increased in GHSR1a KD mice, compared with the blank control,(BC) or SC groups. Values are expressed as the mean ± SD of three independent experiments. *P <* *0.05 versus BC or SC groups. KD, knockdown; BC, blank control; SC, scrambled control.

We next examined the expression of the cell proliferation marker Ki‐67 in tumor tissues from the three groups. The results in Figure 7A showed that positive immunohistochemical staining for Ki‐67 was presented in all of the xenograft tumors. However, the percentage of Ki‐67‐positive‐stained cells was markedly lower in GHSR1a KD xenograft tumors tissues in comparison with blank or scrambled controls. Moreover, cells that were stained into dark brown‐yellow were considered PTEN protein positive. The PTEN‐positive expression of GHSR1a KD mice was higher than other groups (Fig. 7B).

## Discussion

In this study, we explored the functions of ghrelin and its cognate receptor GHSR1a in SW480 colorectal cancer cells. RT‐PCR and Western blot analysis showed that ghrelin and GHSR1a are expressed in normal as well as malignant colorectal cells. Furthermore, the levels of ghrelin and GHSR1 are overexpressed in malignant cells in comparison with normal cells, which is consistent to a previous report 20. The differential expression of ghrelin and GHSR1a was indicative the fact that ghrelin‐GHSR axis might be involved in malignant behavior of colorectal carcinoma through autcorine/paracrine means. In addition, we also provided further evidence for this hypothesis, which was that the ghrelin‐GHSR axis is significantly upregulated in human well‐differentiated and moderately differentiated colorectal cancer tissue samples in contrast to normal colorectal tissues. Nevertheless, as the tumor grade progresses from moderately differentiated to poorly differentiated cancer tissues, the expression of ghrelin and GHSR1a declines. The absence of correlation between the levels of ghrelin or GHSR1a and tumor grades was suggestive the phenomenon that ghrelin‐GHSR axis apparently operates in low‐grade colorectal cancer, but does not functions in high‐grade colorectal cancer.

Previous studies demonstrated that GHSR1a mediates constitutive antiapoptotic activity and increases cell survival when stably expressed in HEK‐293 cells 21. The proproliferative effect of ghrelin can be almost completely abolished by GHSR antagonist in vitro 20. This implies that ghrelin may act through GHSR as an autocrine/paracrine growth factor, promoting the progression of malignant cells. Since ghrelin may also promote cell proliferation by acting through the unknown alternative ghrelin receptor 22, and GHSR1a antagonists may be useful in inhibiting ghrelin‐promoted cell proliferation in colorectal cancer, we aimed to indentify whether ghrelin promotes the proliferation of colorectal cancer cells through GHSR1a. In this study, we successfully constructed the tumor‐specific lentivirus‐mediated shRNA targeting GHSR1a gene and acquired GHSR1a knockdown SW480 cells for the first time. Our results showed that the exogenous acylated ghrelin treatment stimulate the growth of SW480 cells, whereas downregulation of GHSR1a inhibited the growth of SW480 cells with or without ghrelin treatment. This implies that the proproliferative role of ghrelin is acted through GHSR1a to promote cell growth in colorectal cancer cells. However, it is currently obscure whether the inhibition capability that inhibiting the expression of GHSR1a has on cell growth is due to the inhibition of an autocrine ghrelin pathway, along with whether GHSR1a constitutive activity also contributes to colorectal cancer cell growth. This study demonstrated that no effect of the transduction of GHSR1a shRNA is found on the expression of ghrelin in SW480 cells, suggesting that the constitutive activity of GHSR1a plays an important role in cell growth in colorectal cells.

Further approaches were employed to validate the hypothesis that the effects of GHSR1a silencing on the inhibition of colorectal cancer cell growth are sustained in vivo. The stable silencing GHSR1a in SW480 xenografts in nude mice were established. In vivo results demonstrated that the downregulation of GHSR1a in SW480 cells significantly downregulated the expression of Ki‐67 in tumor tissues, leading to a marked reduction in tumor weight. This in vivo study indicated that GHSR1a plays a critical role in the regulation of cell growth and suggested that it may provide a future therapeutic target for colorectal cancer. Nowadays, GHSR antagonists and inverse agonists have been used safely in humans and in in vivo rodent models with satisfied curative effects 23, 24.

PTEN is the first finding in a double specific phosphatase activity of tumor suppressor genes, and it is closely associated with tumorigenesis. Heterozygous loss of PTEN in the mouse resulted in the development of cancer of multiple origins, as well as in a lethal lympho‐proliferative disease 18, 25, 26 . The functional inactivation of PTEN by the regulation of its expression is relevant to many solid tumors. The loss of functional PTEN leads to increased activity of AKT and mammalian target of rapamycin (mTOR) kinase pathways, which promotes both cell survival and proliferation through the phosphorylation as well as inactivation of multiple downstream mediators 27, 28. In addition, it was shown that PTEN exerts positive regulation of p53 level 29. Consistent with such observations, this study found that GHSR1a may target the gene and inhibit the expression of PTEN in mRNA along with protein. GHSR1a shRNA can enhance the level of PTEN to further suppress activated‐AKT signaling as well as an increase in p53, and reduce the ability of proliferation in colorectal cancer cells both in vitro and in vivo. These data showed that the effect of GHSR1a in the progression of colorectal cancer may be activated by the signaling pathway of PTEN/PI3K/AKT.

## Conclusion

In conclusion, our results showed for the first time that the downregulation of GHSR1a by shRNA technology inhibits the growth of colorectal cancer cell line and xenograft tumor through upregulation of PTEN at transcription as well as translation levels. The overexpression of PTEN and p53, along with the inhibition of p‐AKT, thereby, inhibiting cell proliferation in SW480 cells both in vitro and in vivo. This work provided further evidence for the role of the GHSR1a in the functional regulation of the human colorectal cancer. In this way, silencing GHSR1a expression could become a novel therapeutic strategy for colorectal adenocarcinoma prevention and treatment in the future.

## Ethical statement

The ethic committee of the First People Hospital of Yueyang had approved this trial in October, 2012. Informed consent was obtained from all individual participants included in this study. This clinical trial has been registered on Clinical Trials.gov (NCT01336289).

## Conflict of Interest

The authors declare that there is no conflict of interest that could be perceived as prejudicing the impartiality of the research reported.
